# A Novel Cancer Stemness-Related Signature for Predicting Prognosis in Patients with Colon Adenocarcinoma

**DOI:** 10.1155/2021/7036059

**Published:** 2021-10-15

**Authors:** Wei Wang, Congrong Xu, Yan Ren, Shiwei Wang, Chunli Liao, Xiaoyan Fu, Haixia Hu

**Affiliations:** ^1^Department of Gastroenterology, The Second Affiliated Hospital of Fujian Traditional Chinese Medical University, Fuzhou, Fujian 350003, China; ^2^Department of Laboratory, The Second Affiliated Hospital of Fujian Traditional Chinese Medical University, Fuzhou, Fujian 350003, China; ^3^Department of Intense Care Unit, The Second Affiliated Hospital of Fujian Traditional Chinese Medical University, Fuzhou, Fujian 350003, China; ^4^Academy of Integrative Medicine, Fujian University of Traditional Chinese Medicine, Fuzhou, Fujian 350122, China; ^5^Fujian Key Laboratory of Integrative Medicine on Geriatrics, Fujian University of Traditional Chinese Medicine, Fuzhou, Fujian 350122, China

## Abstract

**Objective:**

To explore the cancer stemness features and develop a novel cancer stemness-related prognostic signature for colon adenocarcinoma (COAD).

**Methods:**

We downloaded the mRNA expression data and clinical data of COAD from TCGA database and GEO database. Stemness index, mRNAsi, was utilized to investigate cancer stemness features. Weighted gene coexpression network analysis (WGCNA) was used to identify cancer stemness-related genes. Univariate and multivariate Cox regression analyses were applied to construct a prognostic risk cancer stemness-related signature. We then performed internal and external validation. The relationship between cancer stemness and COAD immune microenvironment was investigated.

**Results:**

COAD patients with higher mRNAsi score or EREG-mRNAsi score have significant longer overall survival (OS). We identified 483 differently expressed genes (DEGs) between the high and low mRNAsi score groups. We developed a cancer stemness-related signature using fifteen genes (including RAB31, COL6A3, COL5A2, CCDC80, ADAM12, VGLL3, ECM2, POSTN, DPYSL3, PCDH7, CRISPLD2, COLEC12, NRP2, ISLR, and CCDC8) for prognosis prediction of COAD. Low-risk score was associated with significantly preferable OS in comparison with high-risk score, and the area under the ROC curve (AUC) for OS prediction was 0.705. The prognostic signature was an independent predictor for OS of COAD. Macrophages, mast cells, and T helper cells were the vital infiltration immune cells, and APC costimulation and type II IFN response were the vital immune pathways in COAD.

**Conclusions:**

We developed and validated a novel cancer stemness-related prognostic signature for COAD, which would contribute to understanding of molecular mechanism in COAD.

## 1. Introduction

As is known to us, colon adenocarcinoma (COAD) is regarded as one of the most frequent malignant tumors in the gastrointestinal system [1–3]. It was reported that there was an increasing incidence of more than 4% in COAD per year all over the world [4]. Cancer heterogeneity results in variable prognosis in patients with COAD [5, 6]. In spite of optimal operative treatment‚ the recurrence rate of COAD is high [6]. Hence, numerous researchers devoted themselves to exploring molecular biomarkers or circulating tumor biomarkers (especially circulating tumor DNA) for identifying minimal residual disease or early relapse [6, 7]. Besides, better characterization of the transcriptomic subtypes and molecular risk stratifications of COAD has greatly improved our understanding of the molecular mechanism of COAD [5, 8, 9]. However, there were no recognized accurate clinical risk stratification methods and biomarkers for COAD [10]. It is of great importance to find out biomarkers or risk stratification models for COAD.

It has been speculated that cancer stem cells (CSCs) played a vital role in expansion, progression, relapse, and therapeutic resistance of solid malignancies [11, 12]. Shiokawa et al. also demonstrated that the slow-cycling subpopulation of CSCs was rather significant in COAD development and chemoresistance [13]. Recently, a novel caner stemness index (named mRNAsi), which was developed by deep learning methods [14], has been extensively researched in various types of cancer, including bladder cancer [15], gastric carcinoma [16], lung cancer [17], and glioma [18]. However, there was not an integrated investigation of COAD stemness features using high-throughput data.

Weighted gene coexpression network analysis (WGCNA) is a recently widespread approach to estimate the distance between genes and identify network topologies [19, 20]. This study utilized WGCNA to investigate cancer stemness features and identify cancer stemness-related genes. Besides, we developed a novel cancer stemness-related prognostic signature for COAD by univariate and multivariate Cox regression analysis. Internal and external validation of this signature was then performed. The relationship between cancer stemness and COAD immune microenvironment was particularly investigated.

## 2. Materials and Methods

### 2.1. Data Source

The transcriptome data and clinical data of COAD were obtained from TCGA database (https://portal.gdc.cancer.gov) [21]. We combined all these data into a matrix file utilizing Perl language (http://www.perl.org/). Ensembl IDs were converted into gene symbols using the Ensembl database (http://asia.ensembl.org/signature.html). We downloaded the gene expression-based stemness index, named mRNAsi, from Malta et al.'s study [14]. Besides, GSE17538 dataset and GSE39582 dataset were downloaded from the GEO database (https://www.ncbi.nlm.nih.gov).

### 2.2. Relationships between Stemness Indices and Clinicopathologic Feature

According to the median value of mRNAsi score, EREG-mRNAsi score, mDNAsi score, and EREG-mDNAsi score, all COAD cases in TCGA database were segmented into low-score group and high-score group, respectively. We then used R package “survival” and “survminer” to explore the difference of overall survival (OS) between the high and low stemness score groups. Besides, the relationships between stemness indices and clinicopathologic features, including age, gender, AJCC stage, T stage, N stage, and M stage, were also investigated using R package “beeswarm.”

### 2.3. Relationships between Stemness Indices and Tumor Microenvironment

Single-sample gene set enrichment analysis (ssGSEA) was performed to calculate the infiltrating score of 16 immune cells and the activity of 13 immune-related function using R package “gsva.” The associations of the stemness indices with immune infiltration cells and immune function activity were then investigated.

There were various components in the tumor microenvironment including tumor cells, immune cells, stromal cells, and extracellular matrix [22]. The ESTIMATE algorithm, which was developed by Yoshihara et al., was recently widely used for exploring tumor microenvironment components [22–24]. We calculated stromal score, immune score, ESTIMATE score, and tumor purity of COAD tumor microenvironment using the ESTIMATE algorithm. The correlation of COAD stemness indices with tumor microenvironment was then explored.

### 2.4. Coexpression Analysis Screening Stemness-Related Genes

The coexpression network targeting these DEGs was established using “WGCNA” package. All paired genes adopted the average linkage method and Pearson's correlation matrices. Moreover, the coexpression similarity matrix was built using the absolute values of the correlations between transcription data. Besides, a power function *a*_*mn*_ = |*c*_*mn*_|^*β*^ (*a*_*mn*_ = adjacency between gene *m* and gene *n*; *c*_*mn*_ = Pearson′s correlation between gene *m* and gene *n*) was used to construct a weighted adjacency matrix. *β*, a soft thresholding parameter, was applied to emphasize strong relations between genes and penalize the weak correlation. Then, an appropriate power of *β* was selected based on the mean connectivity. Next, the network connectivity was measured by converting this adjacency into a topological overlap matrix (TOM). The TOM summed up the adjacent genes for the network gene ratio and calculated the corresponding dissimilarity. Modules were identified using the dynamic tree cut method and named using various colors. The main component for each module was defined as module eigengene (ME). We calculated the correlation between cancer stemness indices and each ME to identify the most significant module. Finally, the modules most highly correlated with mRNAsi and genes in this module were selected for further analysis.

### 2.5. Development of a Cancer Stemness-Related Novel Prognostic Signature

We randomly divided all COAD cases in the GSE39582 cohort into train cohort and test cohort. Univariate and multivariate Cox regression analyses were conducted in the train cohort to develop a cancer stemness-related novel prognostic signature for predicting OS in patients with COAD. Next, all patients in the train cohort were split into two groups, low-risk group and high-risk group, according to median value of the risk score.

### 2.6. Internal and External Validation of This Cancer Stemness-Related Signature

Firstly, we conducted survival analysis and time-dependent receiver operating characteristic (ROC) curve to validate the performance of this cancer stemness-related novel prognostic signature. Then, univariate and multivariate independent prognostic analyses were used to explore whether this signature was an independent risk factor for OS in COAD patients.

Moreover, we utilized the test cohort to perform internal validation for this novel cancer stemness-related prognostic signature. The risk score for each patient in the test cohort was calculated using the risk formula. Next, TCGA cohort and GSE17538 cohort were used as external validation. We calculate the risk score for each case in TCGA cohort and GSE17538 cohort by the risk formula. Survival analysis was then performed.

### 2.7. Associations of the Cancer Stemness-Related Signature with Immune Cell Infiltration and Immune Function

We calculated the infiltrating scores of 16 immune cells and the activity of 13 immune-related pathways of TCGA cohort and GSE39582 cohort using the ssGSEA method. Then, the associations of the cancer stemness-related signature with immune cell infiltration and immune-related pathway activity were explored.

### 2.8. Identification and Validation of Hub Biomarkers in Multidatabase

We selected two genes (including NRP2 or ADAM12) as hub biomarkers for COAD cancer stemness features. The mRNA expression levels between normal and tumor tissues were demonstrated using the GEPIA (http://gepia.cancer-pku.cn/signature.html) database. The UALCAN database (http://ualcan.path.uab.edu/) is a portal for facilitating tumor subgroup gene expression and survival analyses. Promoter methylation levels of hub genes were revealed using the UALCAN database. Survival analysis based on the UALCAN database was performed to evaluate the prognostic value of these two hub biomarkers.

## 3. Results

### 3.1. Associations of COAD Stemness Features with Clinicopathologic Feature

**Figure 1:**
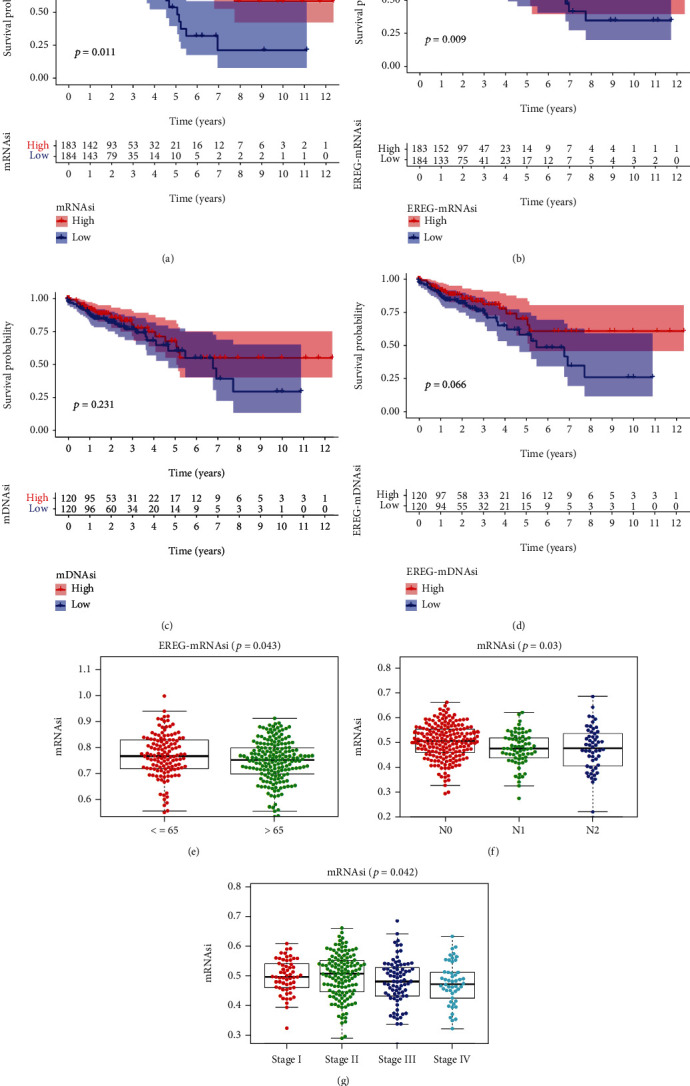
Associations of COAD stemness feature with clinicopathologic features: (a) mRNAsi; (b) EREG-mRNAsi; (c) mDNAsi; (d) EREG-mDNAsi; (e) EREG-mRNAsi and age; (f) mRNAsi and N stage; (g) mRNAsi and stage.

We eventually included 398 COAD samples with complete transcriptome data from TCGA database, 238 COAD cases with transcriptome data from GSE17538 dataset, and 585 COAD cases with transcriptome data from GSE39582 dataset. However, there were merely 385 COAD samples with clinical data in TCGA database and 232 cases with clinical data in GSE17538 dataset. The clinicopathologic data of TCGA cohort, GSE17538 cohort, and GSE39582 cohort are presented in Table 1. Survival curve suggested that the COAD patients with higher mRNAsi score or EREG-mRNAsi score have significantly longer overall survival compared with those with low mRNAsi score or EREG-mRNAsi score. However, there was no significant difference in overall survival between the high and low mDNAsi score and EREG-mDNAsi groups. Moreover, we found that mRNAsi score was significantly associated with AJCC stage and N stage while EREG-mRNAsi score was related to age. However, mDNAsi score was not related to any clinical features. Therefore, we selected mRNAsi to explore the cancer stemness of COAD in this study (Figure 1).

### 3.2. Associations of COAD Stemness Features with Tumor Immune Microenvironments

**Figure 2:**
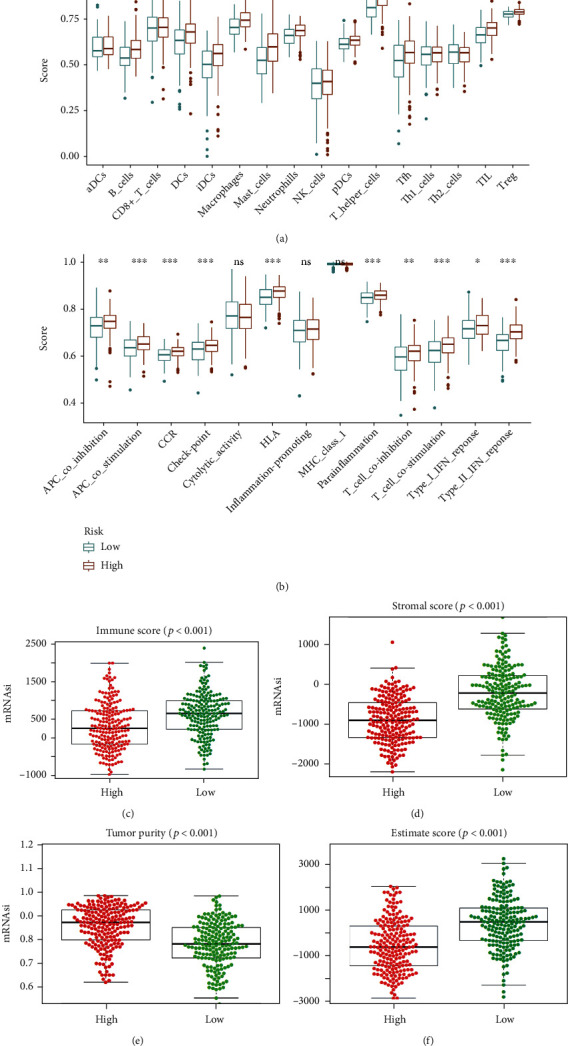
Association of COAD stemness features with tumor immune microenvironment: (a) tumor-infiltrating immune cells; (b) immune function and pathways; (c) immune score; (d) stromal score; (e) tumor purity; (f) ESTIMATE score.

The ssGSEA analysis revealed that higher infiltrating percentages of B cells, DCs, iDCs, macrophages, mast cells, neutrophils, pDCs, T helper cells, Tfh, TIL, and Treg were significantly associated with low mRNAsi score of COAD. Besides, patients with lower mRNAsi have higher scores of immune function or pathways than those with higher mRNAsi, including APC coinhibition, APC costimulation, CCR, checkpoint, HLA, parainflammation, T cell coinhibition, T cell costimulation, type I IFN response, and type II IFN response. We also used the ESTIMATE algorithm to explore the COAD immune environment. The results showed that high mRNAsi score had an association with lower immune score, stromal score, and ESTIMATE score of COAD. However, high mRNAsi score had a positive association with higher tumor purity of COAD (Figure 2).

### 3.3. WGCNA in Identifying Cancer Stemness-Related Genes and Functional Enrichment

**Figure 3:**
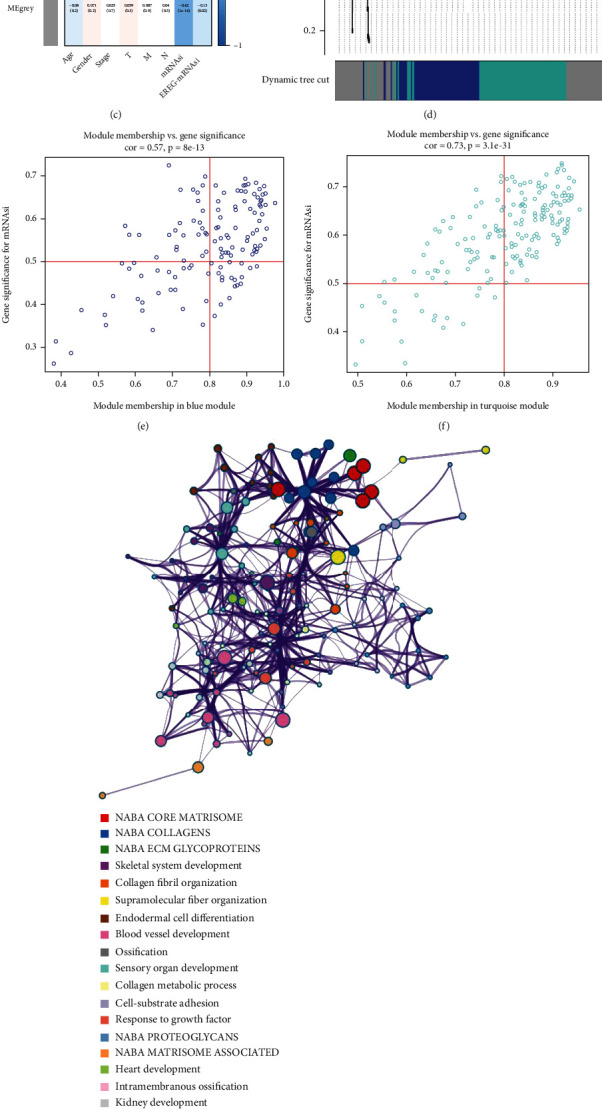
WGCNA identifying key module and genes significantly related to cancer stemness of COAD. Analysis of the scale-free fit signature for various soft-thresholding powers (a). Analysis of the mean connectivity for various soft-thresholding powers (b). Module-trait relationship between module eigengenes and stemness indices (c). Cluster dendrogram (d). Scatter plot of module eigengenes in turquoise module (e). Scatter plot of module eigengenes in turquoise module (f). Functional enrichment analysis for intersection genes (g). *P* value of each gene in the network (h).

There were a total of 386 patients in TCGA database with complete transcriptome data and mRNAsi score. We divided all patients in TCGA database into the low mRNAsi score group (187 cases) and the high mRNAsi score group (199 cases). We identified 483 differently expressed genes between these two groups, including 480 downregulated DEGs and 3 upregulated DEGs. Downregulated DEGs represented genes highly expressed in the low mRNAsi score patients while upregulated DEGs represented genes highly expressed in the high mRNAsi score patients. These genes were imported into coexpression analysis. WGCNA analysis identified a total of 2 coexpression modules, which were shown using various colors. The correlation between each module eigengene (ME) and mRNAsi score was explored to identify the relevant modules. Finally, the turquoise module and blue module were considered as the most significant module and selected for further analysis. We set GS as >0.5 and MM > 0.8 and identified a total of 110 cancer stemness-related genes (Figures 3(a)–3(f)).

Functional analysis demonstrated that these cancer stemness-related genes were mainly enriched in cellular response to vascular endothelial growth factor stimulus, face morphogenesis, kidney development, intramembranous ossification, heart development, NABA MATRISOME ASSOCIATED, NABA PROTEOGLYCANS, response to growth factor, cell-substrate adhesion, collagen metabolic process, sensory organ development, ossification, blood vessel development, endodermal cell differentiation, supramolecular fiber organization, collagen fibril organization, skeletal system development, NABA ECM GLYCOPROTEINS, NABA COLLAGENS, and NABA CORE MATRISOME (Figures 3(g) and 3(h)).

### 3.4. Development of a Novel Cancer Stemness-Based Prognostic Signature

After we excluded those OS time < 30 days, 573 cases in GSE39582 dataset, 355 cases in TCGA database, and 229 cases in GSE17538 dataset were finally included in the development and validation of a novel stemness-based prognostic signature. The GSE39582 dataset has the maximum sample size among these three cohorts. Hence, we used the GSE39582 dataset to develop a cancer stemness-related signature for predicting prognosis of COAD patients. TCGA cohort and GSE17538 cohort were used for external validation. Firstly, we divided all samples in the GSE39582 dataset into train cohort and test cohort. There were a total of 288 cases in the train cohort and 285 cases in the test cohort. Multivariate Cox regression analysis was conducted to develop a novel signature using fifteen cancer stemness-related genes (including RAB31, COL6A3, COL5A2, CCDC80, ADAM12, VGLL3, ECM2, POSTN, DPYSL3, PCDH7, CRISPLD2, COLEC12, NRP2, ISLR, and CCDC8) for prognosis prediction of COAD using the train cohort. The calculation formula of risk score is shown as follows: Risk score = coef1∗Exp1 + coef2∗Exp2 + coef3∗Exp3 + ⋯+coefx∗Expx (Table 2).

### 3.5. Validation of This Novel Cancer Stemness-Based Prognostic Signature

**Figure 4:**
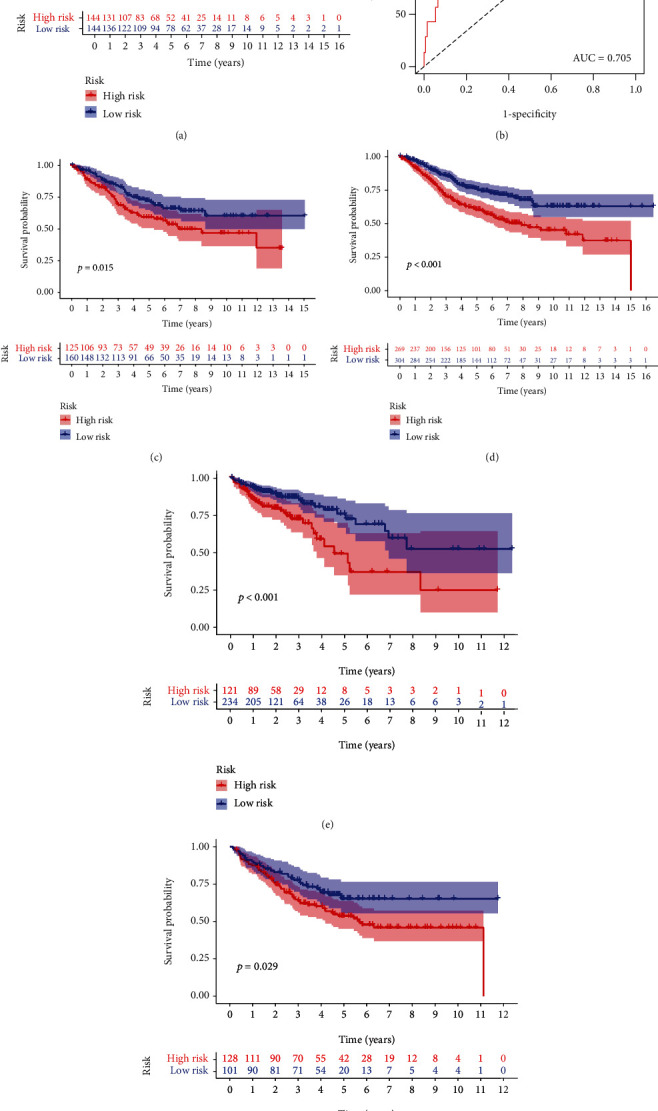
Internal and external validation of a novel cancer stemness-related prognostic signature in COAD patients. (a, b) The survival analysis between the high- and low-risk groups and corresponding area under the ROC curve in the train cohort. The survival analysis between the high- and low-risk groups in the (c) test cohort, (d) whole GSE39582 cohort, (e) TCGA cohort, and (f) GSE17538 cohort.

In the train cohort, the difference in OS between the low-risk and high-risk groups was statistically significant (*P* < 0.001); low-risk score was associated with significantly preferable OS in comparison with high-risk score. The area under the ROC curve (AUC) for OS prediction was 0.705, suggesting the promising value of this novel cancer stemness-based prognostic signature for prognosis prediction of COAD (Figure 4). Univariate independent prognostic analysis showed that age, stage, N stage, M stage, and this prognostic signature were associated with OS of COAD. Multivariate analysis demonstrated that age, N stage, M stage, and this prognostic signature were independent predictors for OS of COAD, indicating the great performance of this signature (*P* < 0.05, Table 3).

**Figure 5:**
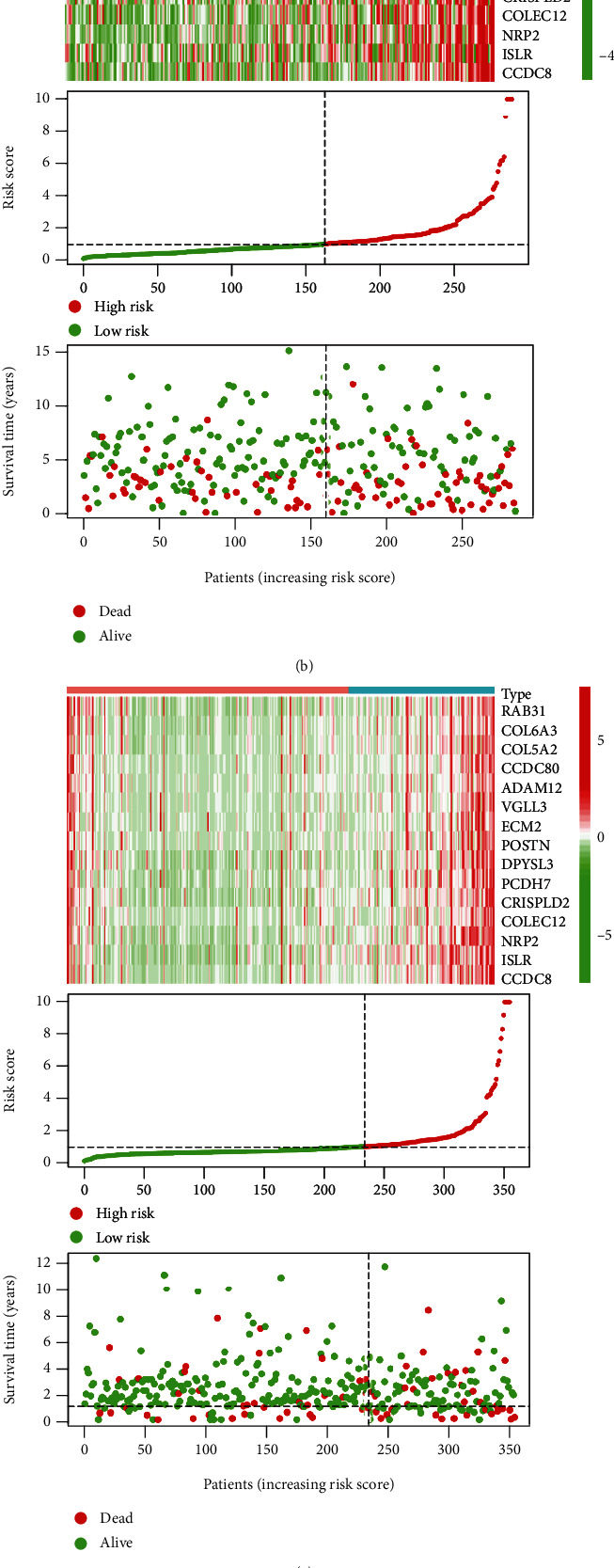
The expression heat map, the distribution of risk score, and survival time of (a) train cohort, (b) test cohort, (c) TCGA cohort, and (d) GSE17538 cohort.

Moreover, internal and external validation was performed. We calculated the risk score of each patient in train cohort, test cohort, TCGA cohort, and GSE17538 cohort. Survival analysis revealed that the difference in OS between the low-risk and high-risk groups was statistically significant in the test cohort (*P* = 0.015), whole GSE39582 cohort (*P* < 0.001), TCGA cohort (*P* < 0.001), and GSE17538 cohort (*P* = 0.029), respectively. The expression heat map, the distribution of risk score, and survival time of train cohort, test cohort, TCGA cohort, and GSE17538 cohort are presented in Figure 5.

### 3.6. Associations of Cancer Stemness-Based Prognostic Signature with Immune Cell Infiltration and Immune Function

**Figure 6:**
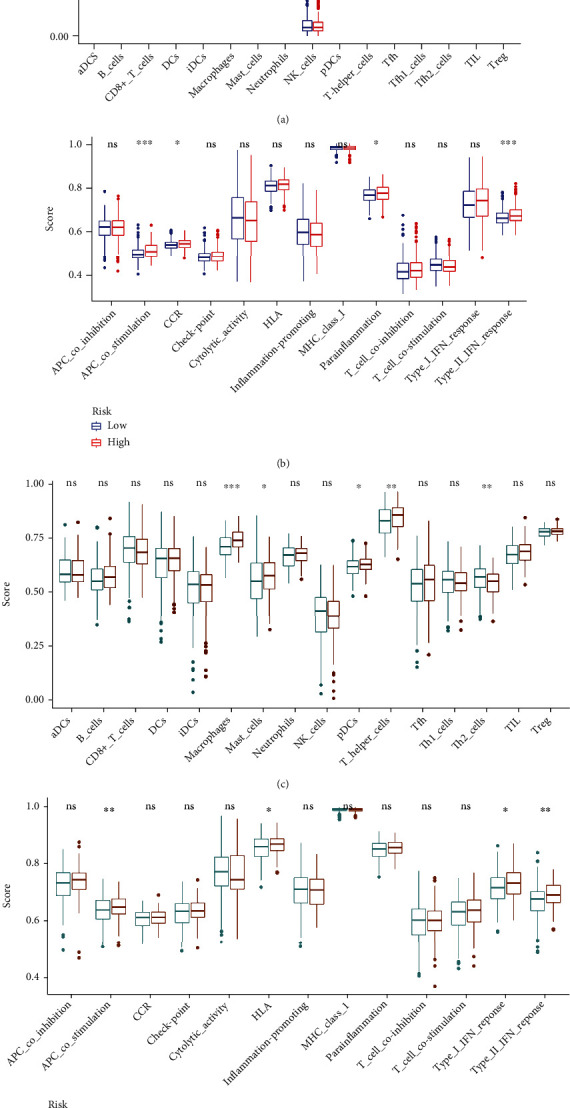
Associations of this signature with immune functions and immune cell infiltration in (a, b) GSE39582 cohort and (c, d) TCGA cohort.

The immune functions and immune cell infiltration were quantified using ssGSEA in TCGA cohort and GSE39582 cohort. For the GSE39582 cohort, the infiltrating proportion of DCs, macrophages, mast cells, neutrophils, and T helper cells in the high-risk group was significantly increased compared with that in the low-risk group; patients with high-risk score have higher scores of immune function or pathways than those with low-risk score including APC costimulation, CCR, parainflammation, and type II IFN response (Figures 6(a) and 6(b)). For TCGA cohort, the infiltrating proportion of macrophages, mast cells, pDCs, and T helper cells in the low-risk group was significantly decreased in comparison with that in the high-risk group; the infiltrating proportion of Th2 cells in the low-risk group was significantly increased in comparison with that in the high-risk group; patients with low-risk score have lower scores of immune function or pathways than those with high-risk score including APC costimulation, HLA, type I IFN response, and type II IFN response (Figures 6(c) and 6(d)). These results showed that macrophages, mast cells, and T helper cells were the vital infiltration immune cells and associated with this cancer stemness-related signature and that APC costimulation and type II IFN response were the vital immune functions and associated with this cancer stemness-related signature.

### 3.7. Validation of Two Hub Genes Significantly Associated with Stemness Features

**Figure 7:**
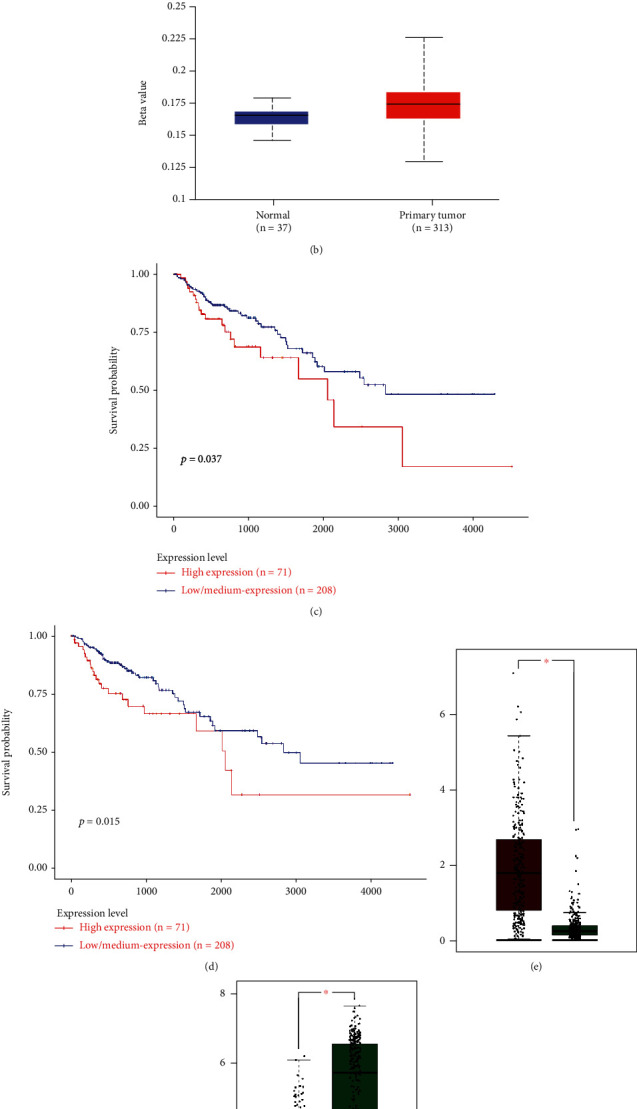
Validation of (a, b) promoter methylation, (c, d) prognostic value, and (e, f) mRNA expression levels of two potential biomarkers.

It was also found that the mRNA expression level of NRP2 was significantly decreased in COAD tissues compared with that in normal tissues while the mRNA expression level of ADAM12 was significantly increased in COAD tissues compared with that in normal tissues. Moreover, the promoter methylation level of NRP2 and ADAM12 was significantly higher in COAD tissues compared with that in normal tissues. Survival analysis reveals that the OS of patients with high expression of NRP2 or ADAM12 was decreased significantly compared with those with low expression (Figure 7).

## 4. Discussions

Numerous studies have revealed that tumors harbored subclones with respect to the sensitivity of various therapy methods, and CSCs were thought to be determining intratumor heterogeneity [25]. Besides, CSCs have been reported to have a vital role in cancer initiation, progression, and chemoresistance, and epigenetic alterations have been regarded as brilliant factors related to CSC phenotype [12]. For example, La Noce et al. revealed for the first time that HDAC2 could serve as a key factor in regulating CSC phenotype and a potential therapeutic target in human osteosarcoma [12]. Over the past decade, colon CSCs also have been reported to be critical in COAD proliferation, invasion, recurrence, and chemoresistance [26]. A deep understanding into cancer stemness features of COAD would contribute to making clinical decision and improving outcomes once CSC targeted therapy is available [27]. In this study, comprehensive bioinformatics analysis was performed to identify stem cell-associated genes for discovering the underlying compounds and developing stemness-related signature for predicting prognosis in COAD. In particular, we explored the association between COAD stemness and tumor immune environment.

In recent years, the tumor immune microenvironment was found to be vital in maintaining tumor stemness [28]. Crespo et al. suggested in their study that introducing/promoting T cell stemness and targeting T cell dysfunctional mechanisms would be of great importance in treating malignant tumors [29]. Su et al. demonstrated that targeting the CD10+GPR77+ cancer-associated fibroblast subpopulation could be an effective therapeutic strategy for CSC-driven solid tumors [30]. In this study, based on the ESTIMATE tumor immune environment evaluation algorithm, we revealed that high mRNAsi score had an association with lower immune score, stromal score, and ESTIMATE score of COAD. Besides, the ssGSEA revealed that macrophages, mast cells, and T helper cells were the vital infiltration immune cells and APC costimulation and type II IFN response were the vital immune pathways in COAD. Previous study also revealed the close associations of COAD stemness with tumor environment and immune cells. Fang et al. demonstrated that IL33 played a vital role in accelerating COAD cell stemness via activating JNK and recruiting macrophages [31]. Hwang et al. indicated that cancer stem-like cell could prime neutrophils for facilitating the occurrence and development of COAD [32]. Yu et al. found that mast cells might be vital in promoting COAD genesis and progression via bidirectional crosstalk [33]. Perez et al. reported that TGF-*β* signaling in Th17 cells was crucial in accelerating IL-22 production and colitis-associated COAD [34]. However, exploration of APC costimulation and type II IFN response in COAD developments is rare. Further research is required.

Moreover, an interesting signature related to cancer stemness of COAD was developed in this study. There have been several prognostic signatures of COAD in previous study. Xu et al. identified a signature using 15 genes based on support vector machine, which could be used to classify COAD patients with various outcomes [10]. Yin et al. established an effective lncRNA signature based on the genome instability as a potential tool for predicting survival of COAD [35]. Nie et al. developed a ferroptosis-related prognostic signature to predict survival of COAD and found that STING might be a new promising immune target [35]. Jiang et al. constructed a vital signature as a novel marker to predict COAD prognosis using lipid metabolism-related genes [36]. Chang et al. used five RNA-binding proteins to develop a superior prognostic and diagnostic signature, which provided new possibilities for individualized treatment of COAD patients [37]. However, no previous studies constructed prognostic signature of COAD from the point of cancer stemness. In this study, we developed a novel cancer stemness-related prognostic signature for COAD using bioinformatics methods for the first time. Univariate and multivariate independent prognostic analyses demonstrated that this cancer stemness-related signature was a vital independent predictor for OS of COAD. Internal and external validation indicated the potential role of this cancer stemness-related signature.

Finally, we identified two cancer stemness-related genes as potential hub biomarkers for COAD, including NRP2 and ADAM12. Fujii et al. found that miR-331-3p could promote the differentiation of keratinocyte through inhibiting NRP2 in cervical cancer cell [38]. Wang et al. revealed that circ-LDLRAD3 was important in promoting the progression of gastric cancer by regulating the miR-224-5p/NRP2 axis [39]. Polavaram et al. demonstrated that targeting NRP2 in prostate cancer might be beneficial in treating bone metastasis [40]. Huang et al. reported that ADAM12 and lnc015192 could serve as a ceRNA by regulating miR-34a in breast cancer [41]. Veenstra et al. proved that ADAM12 could serve as a serum marker for stromal activation and predict chemotherapy sensibility in pancreatic cancer [42]. Shimura et al. found that ADAM12 and MMP-9/NGAL complex in urine could serve as a detective biomarker for gastric cancer [43]. However, researches focusing on the vital role of NRP2 and ADAM12 in COAD were rare. Further exploration into the mechanism of NRP2 and ADAM12 in COAD is required.

However, several inescapable limitations should be noted in this study. The retrospective nature and merely bioinformatics analysis are the major weaknesses of this study. Prospective sequencing data is required. Secondly, these 15 cancer stemness-related genes might be of great importance in COAD tumorigenesis. Further in vitro and in vivo experiment into the underlying mechanism should be performed.

## 5. Conclusion

In our study, we identified differently expressed genes involved in cancer stemness for COAD by comprehensive bioinformatics analysis. More importantly, we successfully developed and validated a novel cancer stemness-related prognostic signature for COAD, which would contribute to further understanding of molecular mechanism in COAD.

## Figures and Tables

**Table 1:** Clinicopathologic data of TCGA cohort and GEO cohort.

Variables	TCGA cohort	GSE39582 cohort	GSE17538 cohort
Age	67.01 ± 12.77	66.95 ± 13.17	64.73 ± 13.43
Gender			
Male	205 (53.2%)	322 (55.0%)	122 (52.6%)
Female	180 (46.8%)	263 (45.0%)	110 (47.4%)
Grade			
G1	—	—	17 (7.3%)
G2	—	—	166 (71.6%)
G3	—	—	30 (12.9%)
G4	—	—	0 (0%)
Unknown	—	—	19 (8.2%)
Stage			
I	66 (17.1%)	28 (17.1%)	28 (12.1%)
II	151 (39.2%)	72 (17.1%)	72 (31.0%)
III	103 (26.8%)	76 (17.1%)	76 (32.8%)
IV	54 (14.0%)	56 (17.1%)	56 (24.1%)
Unknown	11 (2.9%)	0 (0%)	0 (0%)
T stage			
T0	0 (0%)	1 (0.2%)	—
Tis	1 (0.3%)	3 (0.5%)	—
T1	9 (2.3%)	12 (2.0%)	—
T2	68 (17.7%)	49 (8.4%)	—
T3	263 (68.3%)	379 (64.8%)	—
T4	44 (11.4%)	119 (20.3%)	—
Unknown	0 (0%)	22 (3.8%)	—
N stage			
N0	231 (60.0%)	314 (53.7%)	—
N1	88 (22.9%)	137 (23.4%)	—
N2	66 (17.1%)	100 (17.1%)	—
N3	0 (0%)	6 (1.0%)	—
Unknown	0 (0%)	28 (4.8%)	—
M stage			
M0	286 (74.3%)	499 (85.3%)	—
M1	54 (14.0%)	61 (10.4%)	—
Unknown	45 (11.7%)	25 (4.3%)	—
Tumor location			
Distal	—	351 (60.0%)	—
Proximal	—	232 (39.7%)	—
Unknown	—	2 (0.3%)	—
Chemotherapy adjuvant			
Yes	—	240 (41.0%)	—
No	—	326 (55.7%)	—
Unknown	—	19 (3.3%)	—
Survival			
Yes	79 (20.5%)	194 (33.2%)	93 (40.1%)
No	306 (79.5%)	385 (65.8%)	139 (59.9%)
Unknown	0 (0%)	6 (1.0%)	0 (0%)

**Table 2:** Multivariate Cox regression analysis to develop a cancer stemness-related prognostic signature for colon adenocarcinoma.

Gene	Coef	HR	HR.95L	HR.95H	*P* value
RAB31	-0.2497624	0.77898584	0.68102434	0.89103856	0.00027
COL6A3	-0.09365407	0.91059771	0.85538562	0.96937355	0.003339
COL5A2	0.10028935	1.10549074	1.03561084	1.18008593	0.00261
CCDC80	-0.13118117	0.87705886	0.74191702	1.03681709	0.124419
ADAM12	-0.28676898	0.75068513	0.60566585	0.93042751	0.008835
VGLL3	0.40961615	1.5062395	0.93041462	2.438437	0.095609
ECM2	-0.2827304	0.75372297	0.50851665	1.11716758	0.159089
POSTN	0.04015551	1.04097264	1.00999719	1.07289808	0.009177
DPYSL3	-0.04823193	0.95291275	0.89170774	1.01831875	0.154443
PCDH7	0.44823424	1.56554537	0.98716952	2.48278765	0.05677
CRISPLD2	0.15000434	1.16183929	1.00084539	1.34873033	0.048716
COLEC12	0.35954641	1.43267942	0.98120917	2.09187844	0.06264
NRP2	0.28532447	1.33019357	1.08404781	1.6322296	0.006277
ISLR	0.03457653	1.03518124	0.99407226	1.07799025	0.094446
CCDC8	0.49774993	1.64501571	1.02341561	2.64416202	0.039826

**Table 3:** Univariate and multivariate independent prognostic analyses.

ID	Univariate				Multivariate			
	HR	HR.95L	HR.95H	*P* value	HR	HR.95L	HR.95H	*P* value
Gender	1.314912	0.826428	2.09213	2.48*E* − 01	—	—	—	—
Age	1.02788	1.006926	1.049271	0.008877	1.027613	1.008673	1.046909	0.004107
Stage	2.034221	1.419878	2.914374	0.000108	0.670314	0.366029	1.227554	0.195043
T	1.476774	0.960467	2.270628	7.57*E* − 02	—	—	—	—
N	1.558316	1.182927	2.052831	1.61*E* − 03	1.72189	1.105289	2.682471	0.016281
M	6.696278	3.412833	13.13869	3.21*E* − 08	10.04966	3.454523	29.23581	2.28*E* − 05
Tumor location	1.073448	0.667182	1.727101	7.70*E* − 01	—	—	—	—
Chemotherapy	0.981772	0.613897	1.570095	0.938789	—	—	—	—
Risk score	1.802561	1.510596	2.150956	6.34*E* − 11	1.644224	1.365092	1.980431	1.62*E* − 07

## Data Availability

All data generated or analyzed during the present study were downloaded from TCGA database and GEO database.
